# Gold Nanoparticles and Graphene Oxide Flakes Synergistic Partaking in Cytosolic Bactericidal Augmentation: Role of ROS and NOX2 Activity

**DOI:** 10.3390/microorganisms9010101

**Published:** 2021-01-05

**Authors:** Osamah Al Rugaie, Majid Jabir, Rua Kadhim, Esraa Karsh, Ghassan M. Sulaiman, Salman A. A. Mohammed, Riaz A. Khan, Hamdoon A. Mohammed

**Affiliations:** 1 Department of Basic Medical Sciences, College of Medicine and Medical Sciences, Qassim University, Unaizah, P.O. Box 991, Al-Qassim 51911, Saudi Arabia; o.alrugaie@qu.edu.sa; 2 Department of Applied Sciences, Division of Biotechnology, University of Technology, Baghdad 35010, Iraq; ruajali@yahoo.com (R.K.); esraa.h97@yahoo.com (E.K.); 3 Department of Pharmacology and Toxicology, College of Pharmacy, Qassim University, Qassim 51452, Saudi Arabia; 4 Department of Medicinal Chemistry and Pharmacognosy, College of Pharmacy, Qassim University, Qassim 51452, Saudi Arabia; ri.khan@qu.edu.sa (R.A.K.); ham.mohammed@qu.edu.sa (H.A.M.); 5 Department of Pharmacognosy, Faculty of Pharmacy, Al-Azhar University, Cairo 11371, Egypt

**Keywords:** gold nanoparticles, graphene oxide flakes, phagocytosis, bactericidal, NOX2, *Staphylococcus aureus*, *Escherichia coli*, bone marrow-derived macrophages

## Abstract

Gold nanoparticles (GNPs) and graphene oxide flakes (GOFs) exerted significantly (*p* < 0.0001) supportive roles on the phagocytosis bioactivity of the immune cells of phagocytic nature against the Gram-positive and Gram-negative human pathogenic bacteria *Staphylococcus aureus* and *Escherichia coli*. Under experimental conditions, upon bacterial exposure, the combined GNPs and GOFs induced significant clearance of bacteria through phagosome maturation (*p* < 0.0001) from time-points of 6 to 30 min and production of reactive oxygen species (ROS, *p* < 0.0001) through the NADPH oxidase 2 (NOX2, *p* < 0.0001)-based feedback mechanism. The effects of the combined presence of GNPs and GOFs on phagocytosis (*p* < 0.0001) suggested a synergistic action underway, also achieved through elevated signal transduction activity in the bone-marrow-derived macrophages (BMDM, *p* < 0.0001). The current study demonstrated that GNPs’ and GOFs’ bactericidal assisting potentials could be considered an effective and alternative strategy for treating infections from both positive and negative bacterial strains.

## 1. Introduction

Various nanoparticles (NPs) are employed extensively in nanobiotechnology and biomedicine fields due to their unique physicochemical features of size, shape, and other physicochemical characteristics, including their reactivity toward biocomponents. NPs function as antigen carriers [1], imaging reagents, molecular tags [2], therapeutic, diagnostic, and theranostic entities, and also as part of simultaneous delivery modules [3,4,5]. The multifunctional core-shell nanoplatforms made from gold nanoparticles (GNPs) and graphene oxide flakes (GOFs) for miRNA delivery and their study on the delivery and release profile were reported [6,7]. Notably, as NPs are foreign to the body, macrophages, characterized by plasticity and heterogeneity [8], have to play crucial roles in in vivo situations to recognize, process, and clean-off NPs [9]. When activated, macrophages exert M1 or pro-inflammatory and M2 as anti-inflammatory phenotypes [10], with M1 exerting crucial effector cells during the resistance responses against intracellular pathogens and tumour growths [11,12].

In contrast, M2 cells are more involved in bio-reactions, such as immunosuppression, induction of tissue remodelling, and tumour progression [13]. M1 and M2 states showed different capacities for the uptake of nanoparticles of various types [14], and, in parallel, NPs can join the micro-milieu stimuli and contribute to prime macrophages to polarize into one stage or of the other [15,16,17,18]. Further, various types of NPs can affect macrophage polarization and reprogram in different manners [19]. This interaction between nanoparticles and these macrophagic cells of differential states caught the nanotoxicology and medicinal application fields’ attention regarding the nanoparticles and other involved nanoentities [20]. The modulation of the in vivo biological influences of the NPs and design of therapies and therapeutic regimes that are NP-based require a deeper understanding of the roles that these nanoscale particles play in the polarization of macrophages [21,22]. Gold NPs (GNPs) were investigated extensively in immunotherapy and vaccine development due to their distinct characteristics and marked progress in the field. GNPs under 20 nm in size facilitate their preferential intracellular accumulation, particularly within immune system cells, and were observed to perform their regular function of interacting with foreign entities and encountered substances.

Nonetheless, as a result of enhanced permeability and retention, GNPs also undergo passive accumulation into the leaky and immature vasculature, in addition to solid tumours [23]. Various factors influence the immunomodulatory activities of GNPs, e.g., specific targeting of certain cells or organs, the capacity of up taking cells, higher release kinetics, and facilitated and fast systemic clearance of nanoentities [24]. The physicochemical features of the GNPs need to be precisely controlled in shape, size, and properties to achieve the higher capability of these nanoentities to reach targeted cells, which certainly lead to more successful applications of these nanoparticles in benefiting immunotherapy and vaccine development. NP tolerance and immunity are induced by dendritic cells (DCs), as well as other antigen-presenting cells (APCs) and, exactly for this reason, the targeting of DCs is crucial during GNP engagement to enhance the desired levels of immunomodulatory responses.

The graphene family materials, including graphene oxide (GO), reduced/functionalized graphene oxide (rGO), graphene quantum dots, graphene nanoribbons, 3D (three-dimensional) graphene foam, and graphene nanopores, all are known to exert enormous effects in various biomedical applications. These materials were tried as antibacterial [25,26], anticancer [27,28], drug delivery [29,30,31], bio-sensing [32,33,34], and bio-imaging [35,36] processes owing to their differential interactions with biosystems and biomolecules under in vivo and in vitro conditions. This potential ascended from the characteristic physical, chemical, and mechanical properties of NPs and flakes to participate in biological processes at subcellular, cellular, and upcellular levels and scales. The high surface-to-volume ratio, effective larger surface area at the nanoscale dimensions, ability to surface functionalize, and the possession of remarkable colloidal stability in the aqueous medium compared to the characteristics and stability of the pristine mono- and multilayered graphenes [37,38,39,40] makes the material more desirable. GO, and rGO possess different physical and chemical characteristics, including differences in solubility, dispensability, lateral dimensions, sheet size, and the degrees and extents of their participation in redox reactions. Also, cellular uptake and biodegradation can easily be manipulated by using different reagents to carry out oxidation and reduction reactions [41,42,43]. The differences in bioactivities levels of GO and rGO are reported in several types of bacteria, as well as in various cancer and noncancerous cells [44,45,46].

The current study aimed to understand the roles of GNPs and GOFs on phagocytic activity in dealing with the human pathogenic bacteria *Staphylococcus aureus (S. aureus)* and the *Escherichia coli (E. coli)*.

## 2. Materials and Methods

### 2.1. Materials

The gold nanoparticle (GNPs, Cat.NO.741965-25ML) and graphene oxide flakes (GOFs, Cat. NO. 763713-250MG) were purchased from Sigma (Milwaukee, WI, USA). The carbon contents in GOFs were between 42% and 52% for the product according to the specification supplied by Sigma. The GOFs were used after sonication in deionized water to exfoliate into monolayers as an aqueous dispersion. Deionized water, a strong repulsive media for the GOFs, ensured that aggregation did not persist, and the GOFs were available as single-layer flakes. GNPs and GOFs were mixed at a concentration of 10 µg/mL each. Moreover, the samples were freshly prepared and utilized immediately. GNPs and GOFs were characterized by UV-visible spectroscopy in a quartz cuvette and Scanning Electron Microscopy (SEM) and Transmission Electron Microscopy (TEM), respectively. The GNPs were of 21.33 ± 4.51 nm in diameter size.

### 2.2. Bone Marrow-Derived Macrophages (BMDMs)

Male C57/BL6 mice (7–8 weeks old) were used as the source for isolation of primary BMDMs, following the previously reported method [47]. All experiments using mice were performed in accordance with the US National Institute of Health (NIH) Guide for the Care and Use of Laboratory Animals (NIH Publication No. 86–23, revised in 1996) and were approved by the Animal Care and Ethics Committee at Biotechnology Division, Applied Sciences Department, University of Technology, Baghdad, Iraq.

### 2.3. Blood Sampling and Preparation

#### 2.3.1. Blood Samples

Fresh samples of blood were taken from 10 healthy donors and collected in heparin-coated tubes as described [48] based on Helsinki’s declaration and regulation of 1975 as a statement of ethical principles. All the methods were carried out in accordance with all the relevant guidelines and regulations for working with human origin samples. Permission was obtained from the hospitals of the medical city, Baghdad, Iraq. The institutional ethics committee approved all experimental protocols of the University of Technology, Baghdad, Iraq (Ref. No. AS 20-8-01-2020). The participants were informed about the study before the collection of any data or samples. Informed consent was obtained from the study participants.

#### 2.3.2. Human Neutrophils Isolation

Neutrophil isolation medium (5.0 mL) was taken into a centrifuge tube; the blood sample (5.0 mL) was carefully added, and centrifuged (2000 RPM, 10 min, 4 °C). The detailed procedure was followed according to the reported protocol [49]. The cells were counted and adjusted to 1 × 10^6^/mL, and pretreated with GNPs and GOFs (1 h, 10 µg/mL each), followed by infection with *S. aureus* (multiplicity of infection (MOI)—1:100) and incubation (37 °C, 30 min). The phagocytic index was calculated using the following equation:

(1) $$\;\mathrm{Phagocytic}\;\mathrm{index}\;=\frac{a}{a-b}\;$$

where *a* refers to nonphagocytic cells and *b* refers to phagocytic cells.

### 2.4. Intracellular Bacterial Killing Assay

*S. aureus* and *E. coli* were cultured (37 °C) in Lysogeny Broth (LB) broth until the mid-log phase (optical density (OD), 0.4–0.6). Following centrifugation (3500× *g*, 15 min, 4 °C), sterile Phosphate Buffered Saline (PBS) was used to wash the pellets 3 times. The concentration of the re-suspended bacteria was determined by measuring OD at 550 nm. The BMDMs were used alone or pretreated with GNPs, GOFs, and combined GNPs and GOFs mixture at 10 µg/mL each. The cells were then infected with *S. aureus* or *E. coli* at M.O.I. 1:50 and incubated at 37 °C for 60 min. The BMDMs were treated with lysis buffer. To determine total and extracellular bacterial killings, serial dilutions (10×) of the lysates were incubated with Luria-Bertani (LB) agar plates (37 °C, 24 h). Values of the total and extracellular bacterial killings were used to derive the intracellular bacterial killing values.

### 2.5. Determination of pH of Phagosomes

The luminal pH of the phagosomes was measured according to the previous method [50]. Briefly, double-labelling of the heat-killed isolates of *S. aureus* and *E. coli* were performed using 5 mg/mL carboxy-fluorescein-succinimidyl ester (SE), a fluorescent probe with sensitivity to pH; Molecular Probes, Eugene, OR, USA, and 10 mg/mL carboxy-tetraethyl-rhodamine-SE (a fluorescent probe with no sensitivity to pH; Molecular Probes, Eugene, OR, USA). Next, a pulse process for the isolated BMDMs was conducted with the labelled bacteria (MOI = 1:50; 30 min) and pursued at 37 °C for each of the mentioned periods, the presence and absence of GNPs, and GOFs. Phagosome pH was extracted using the ratio of fluorescein/rhodamine fluorescence using a standard curve.

### 2.6. Assay of Phagosome/Lysosome Fusion

The BMDMs were isolated and plated into 4-well chamber-slides at a concentration of 1 × 10^5^ cells/mL in RPMI-1640 media. After 10 h, the BMDMs were pretreated with GNPs and GOFs at a concentration of 10 µg/mL each for 1 h. The Lysotracker Red was used to load the cells (25 nM, 37 °C, 60 min), then incubated with Fluorescein isothiocyanate (FITC)-conjugated *S. aureus* (MOI—1:50, 2 h). The Lysotracker Red was added during infection time. Sterile, cold PBS was used to wash the cells five times, followed by fixation with 4% paraformaldehyde and nuclei staining using 4′,6-diamidino-2-phenylindole (DAPI). Samples were mounted and examined with a fluorescent microscope (Olympus, Tokyo, Japan). Unfused phagosomes containing FITC-bacteria appeared with a green stain, whereas the lysosomes labelled with Lysotracker appeared red. The fusion of these phagosomes and lysosomes seemed to be yellow as a result of the fusion of the two labelled fluorochromes.

### 2.7. Phagocytosis of pHrodo E. coli BioParticles by BMDMs

The BMDM cells were plated in a 4-well plate. The cells were pretreated with GNPs and GOFs at a concentration of 10 µg/mL each. pHrodo-particles were mixed in 1 × PBS (pH 7.4), vortexed, and 20 µL of the solution was added to the cells. Phagocytosis was allowed for 2 h, and the cells were fixed and stained with DAPI; images were captured using a Zeiss confocal microscope at 400×. The blue colour represented the cells’ nucleus, and the red dots showed the *E. coli-pHrodo* constituent in phagocytes.

### 2.8. NADPH Oxidase Assay

Lucigenin (bis-N-methyl acridinium nitrate; Sigma-Aldrich, USA) was used to determine the NADPH oxidase (NOX2) activity of BMDMs. Briefly, an incubation step (37 °C for different periods) was performed for BMDMs with heat-killed and opsonized isolates of *S. aureus* and *E. coli* (each separately with phagocytes/bacteria ratio = 1:50) in the presence and absence of GNPs and GOFs at the concentration of 10 µg/mL each.

### 2.9. Statistical Analysis

The data ere expressed as the mean ± SE. All experiments were carried out in triplicate. Differences between groups were analyzed using one-way ANOVA for the phagocytic index, intracellular killing, and phagocytosis of *pHrodo E. coli BioParticles,* while two-way ANOVA was used for phagosomal maturation, ROS, and NOX2 followed by a posthoc test using Tukey’s multigroup comparisons on GraphPad Prism 8.0.2, San Diego, FL, USA. The data were considered significant if *p* < 0.05 [51].

## 3. Results and Discussion

### 3.1. Characterization of the GNPs and GOFs

**Figure 1:**
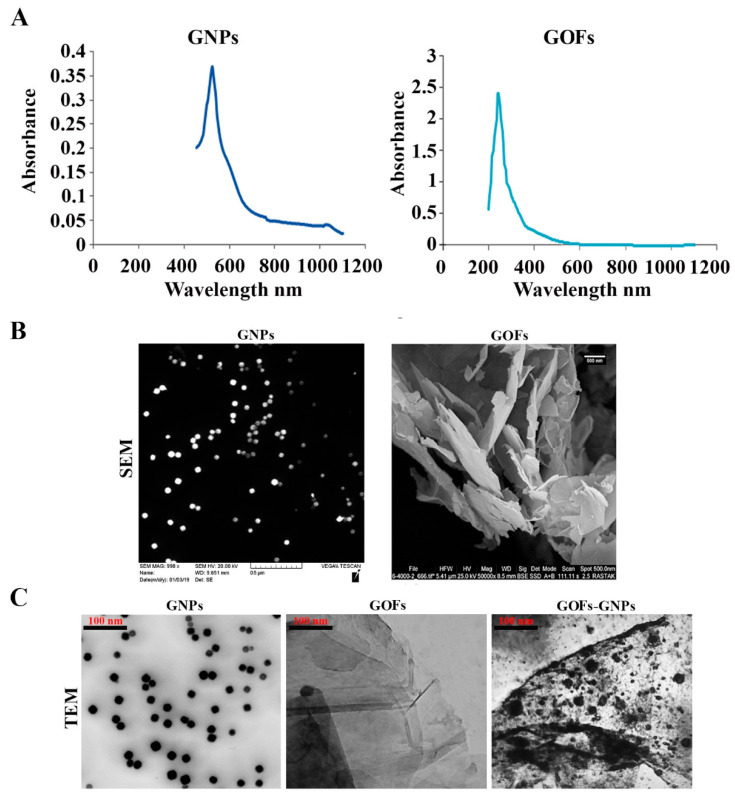
(**A**) UV-VIS spectroscopy analysis and (**B**) Scanning Electron Microscope (SEM) images of gold nanoparticles (GNPs) and graphene oxide flakes (GOFs); (**C**) Transmission Electron Microscope (TEM) images of GNPs, GOFs (exfoliated, single-layer sheets), and a mixture of GNPs and GOFs.

The GNPs and GOFs were analyzed by UV-visible (UV-VIS) spectroscopic method and were found to exhibit the λmax absorption values at 528 nm and 230 nm for the GNPs and GOFs, respectively (Figure 1A). The morphology of the gold nanoparticles and graphene oxide was studied using SEM (Figure 1B) and was found to be spherical for the GNPs with a size of 21.33 ± 4.51 nm and flakes for the GOFs. TEM results are shown in Figure 1C. The GNPs were dispersed on the surface of GOFs. Most of the GNPs were located on the surface of the GOFs and did not protrude from the surface, indicating very strong interactions between GOFs and GNPs. GOFs in the aqueous media ruled out any aggregations of GOFs, which are lipophilic. The Raman spectra confirm the difference between the single- and multilayer GO sheets by exhibiting single peaks at ~2679 cm^−1^ [52,53,54].

### 3.2. GNPs and GOFs Increase the Phagocytic Index of Human Neutrophils

**Figure 2:**
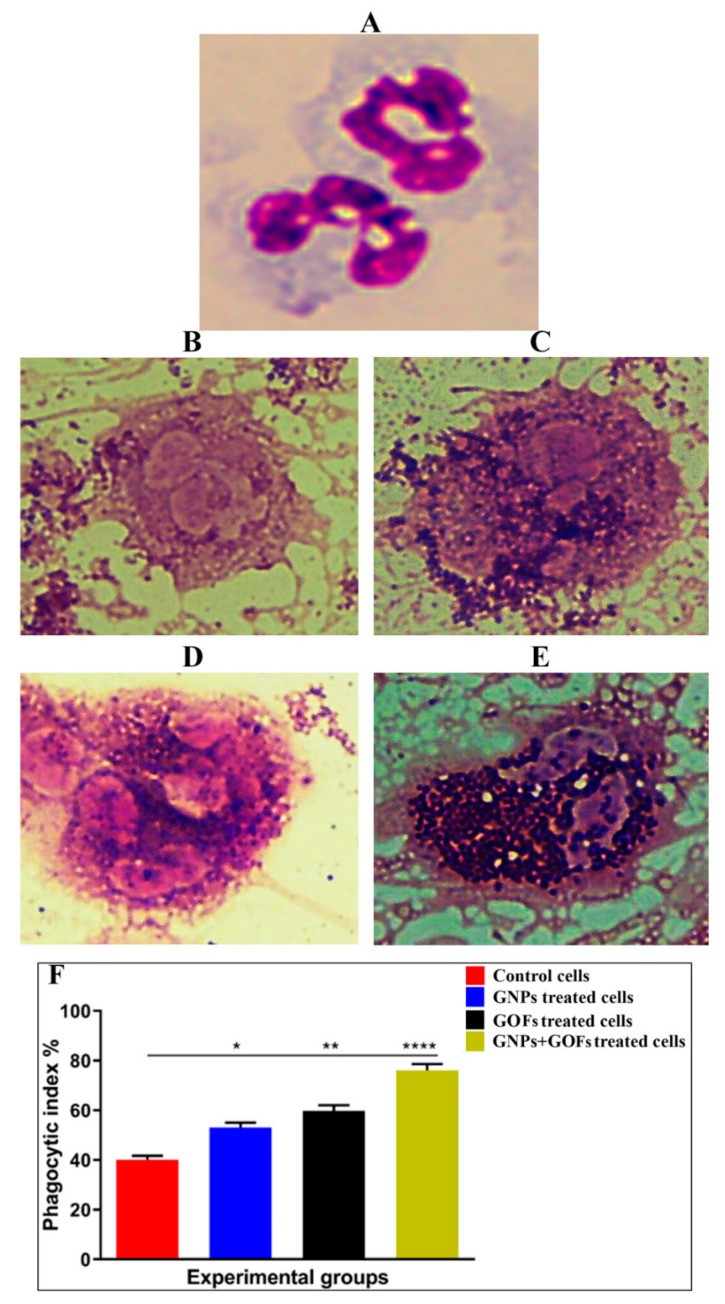
GNPs and GOFs increase the phagocytic index of human neutrophils. (**A**) Control uninfected cells. (**B**) Infected cells with *Staphylococcus aureus*. (**C**) GNP-pretreated cells at a concentration of 10 µg/mL infected with *Staphylococcus aureus*. (**D**) GOF-pretreated cells at a concentration of 10 µg/mL infected with *Staphylococcus aureus*. (**E**) GNP- and GOF-pretreated cells at a concentration of 10 µg/mL each infected with *Staphylococcus aureus*. The images were visualized at 100×. (**F**) Phagocytic index percentage. Data are represented as mean ± SE. Statistical significance by one-way ANOVA (*p* < 0.0001) followed by posthoc testing using Tukey’s multi group comparison: * *p* = 0.01, ** *p* = 0.001, **** *p* < 0.0001 indicate statistically different from the control.

GNPs and GOFs were tested for their capability to induce human neutrophils (Figure 2A–E). The results showed an increase in phagocytic human neutrophil activity, followed by infection with *S. aureus* after adding the GNPs and GOFs at a concentration of 10 µg/mL each (Figure 2F). The nanoparticles increased the phagocytic cells’ activity known to contribute to IL-8 production [55]. An increase indicated the nanoparticles’ capacity as immune-modulators by increasing the phagocytic cells’ activity to engulf the bacteria. Phagocytic cells, such as human neutrophils, were previously shown to possess the ability to induce cytokines, such as IL-8, after treatment with silver nanoparticles (AgNPs) [56].

The entry of the GNPs and GOFs demonstrated significantly (*p* < 0.0001) increased phagocytosis activity at the cellular scale experiment compared to the control (Figure 2F). The biological activity details on the antibacterial action of the GOFs, as shown in Figure 2D, did not exhibit any significant morphological changes for the GOFs. According to reference [57], bacteria and GOF interactions are expected to reduce GOFs, resulting in a lesser oxygen component ratio in the GOFs. However, the quantitation of the carbon could not be estimated at the cellular level. The interaction of the bacteria and GOFs affects the morphology and chemical contents of the GOFs. The GOFs are biocompatible for the bacteria, and the reduced GOFs were expected to increase the antibacterial action slightly. The sheets also work as adsorption sites for bacteria, thereby inhibiting the proliferation of bacteria in cellular compartments.

### 3.3. GNPs and GOFs Increase Bacterial Intracellular Killings

**Figure 3:**
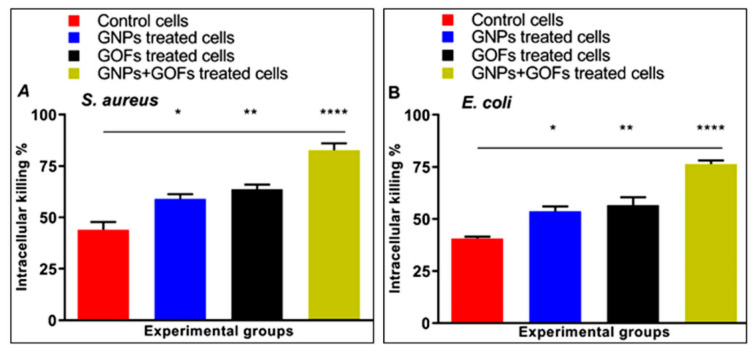
GNPs and GOFs increased the intracellular killing of ingested bacteria by bone-marrow-derived macrophages (BMDMs). Data are represented as mean ± SE. Statistical significance by one-way ANOVA (*p* < 0.0001) followed by posthoc testing using Tukey’s multi group comparison with control cells: (**A**) * *p* = 0.03, ** *p* = 0.007, **** *p* < 0.0001 indicate statistically different for *S. aureus*, (**B**) * *p* = 0.02, ** *p* = 0.007, **** *p* < 0.0001 indicate statistically different for *E. coli*.

The numbers of internalized *S. aureus* and *E. coli* in BMDMs were tested following infection in the presence or absence of GNPs and GOFs to gauge the activity of the phagocytosis process, intracellular killings, and comparison between the two conditions of the presence and absence of the nanoparticles. Both the GNP- and GOF-pretreated phagocytes showed a significant increase in their intracellular killings of the ingested, live *S. aureus*, and *E. coli*, as exhibited in Figure 3A,B, while combined GNP- and GOF-treated cells demonstrated maximum significant increment (*p* < 0.0001). Thus, the GNP and GOF-pre-treated phagocytic cells demonstrated an increased bactericidal response to Gram-positive and Gram-negative strains.

### 3.4. The GNPs and GOFs in Phagosome Maturation

**Figure 4:**
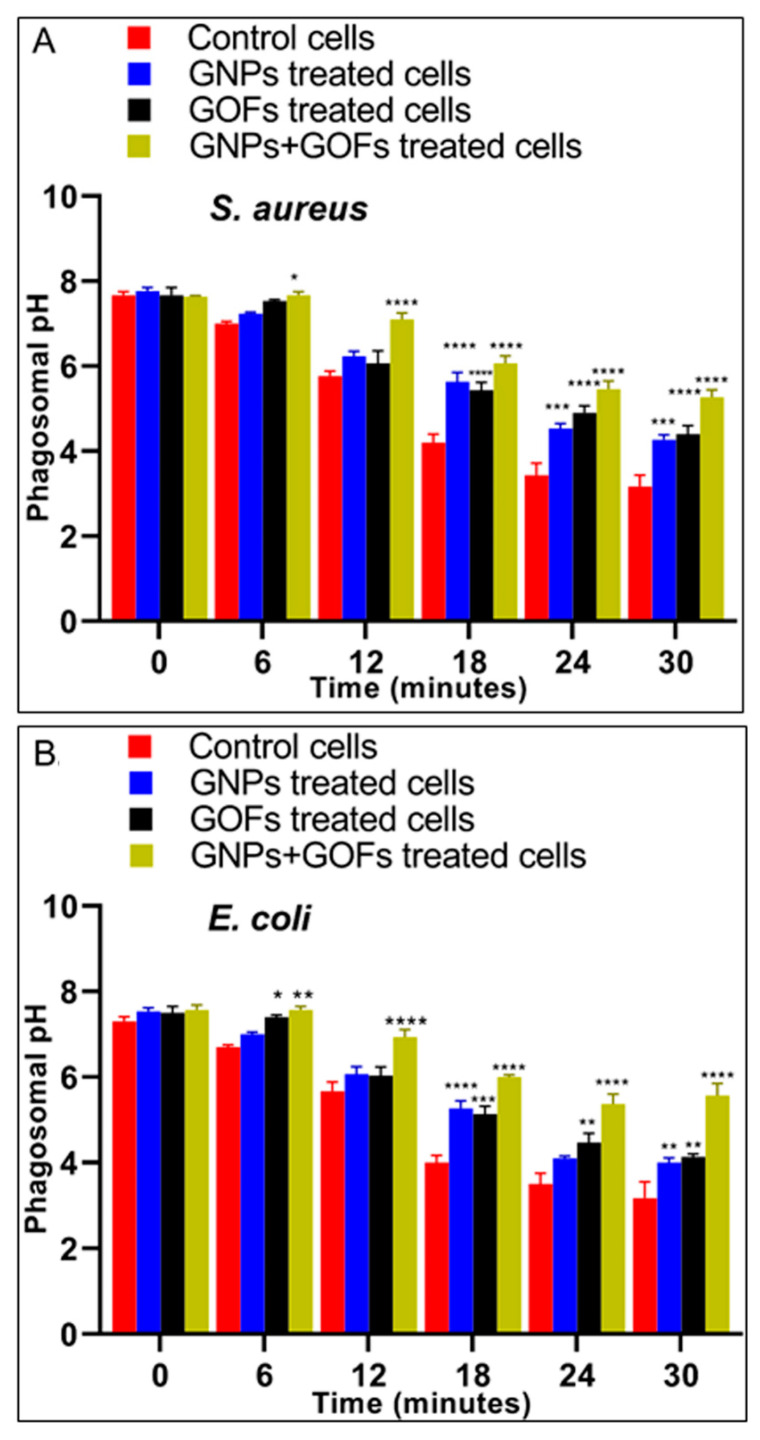
GNPs and GOFs induce phagosome maturation in BMDMs. Phagosomal pH was measured in control, untreated BMDMs, pretreated with GNPs and GOFs as indicated, after being chased with fluorescent probe-coupled *Staphylococcus aureus* or *Escherichia coli*. Data are represented as mean ± SE. Statistical significance by two-way ANOVA (*p* < 0.0001) followed by posthoc testing using Tukey’s multi group comparison with control cells: * *p* = 0.03, *** *p* = 0.002, **** *p* < 0.0001 indicate statistically different for (**A**) *S. aureus* while * *p* = 0.03, ** *p* < 0.01, *** *p =* 0.002, **** *p* < 0.0001 indicate statistically different for (**B**) *E. coli*.

**Figure 5:**
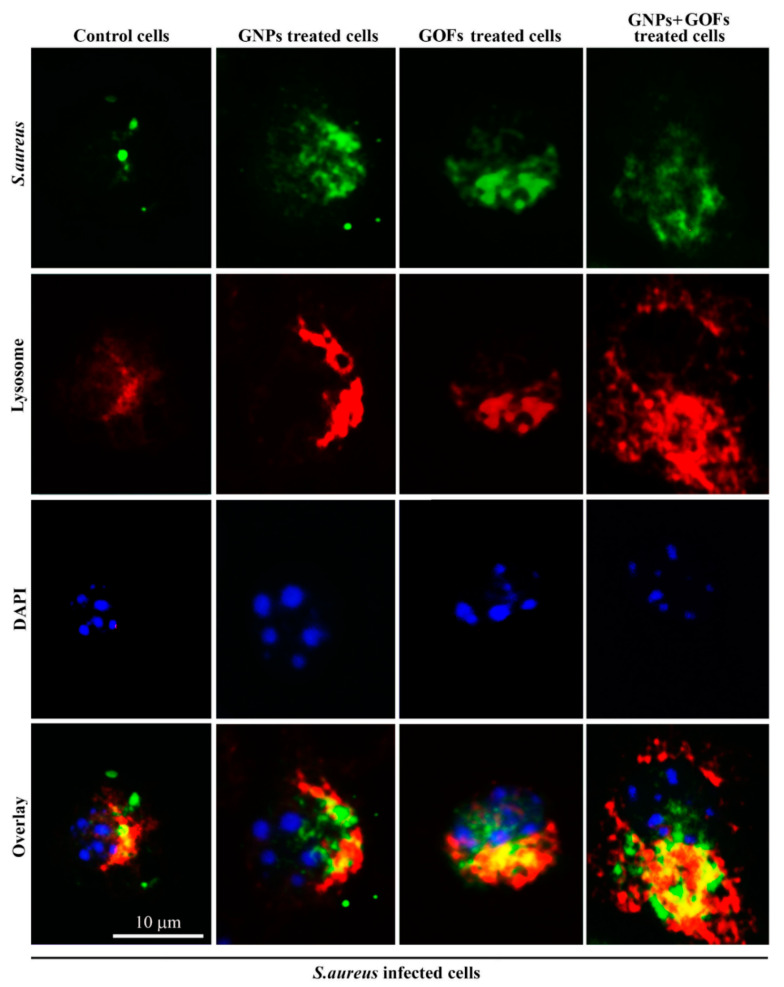
GNPs and GOFs enhanced co-localization between Lysotraker and Fluorescein isothiocyanate (FITC)-conjugated *Staphylococcus aureus*. Co-localization appeared in yellow, lysosomes labelled with Lysotracker appeared red, cell nuclei were stained with 4′,6-diamidino-2-phenylindole (DAPI) appeared in blue, scale bar, 10 µm. The scale bar is identical for all, as the magnifications are the same for the images.

Following the ingestion of bacteria, acidification by phagosomes and the fusion of phagosomes and lysosomes are two main characteristics of phagocyte maturation [58]. Our results showed a significant increase in phagosomal acidification of the GNP- and GOF-pretreated BMDMs following the ingestion of *S. aureus* and *E. coli*, as compared with the control BMDMs (Figure 4). Further loading of BMDMs with Lysotracker red, which serves in labelling the late endosomes/lysosomes selectively, allowed monitoring of the maturation events of the *S. aureus*-FITC ingesting phagosomes through testing their overtime capability of co-localization with Lysotracker red. The results demonstrated the occurrence of this co-localization at 30 min in BMDMs pretreated with GNPs and GOFs. In parallel, most *S. aureus*-FITC entities showed less co-localization with the Lysotracker in control BMDMs (Figure 5).

Moreover, following the ingestions of *S. aureus*, an increase in phagosomal acidification in GNP- and GOF-pretreated BMDMs was observed. Again, the nanoparticle-pretreated macrophages demonstrated significantly increased phago-lysosome fusions in response to *S. aureus* and other bacterial challenges, such as *E. coli*. These results, therefore, demonstrated that BMDMs, which were pretreated with GNPs and GOFs, possessed improved phagosome maturation following the ingestion of *S. aureus* and *E. coli*.

### 3.5. GNPs and GOFs Increase Phagocytosis of pHrodo E. coli Bioparticles by BMDMs

**Figure 6:**
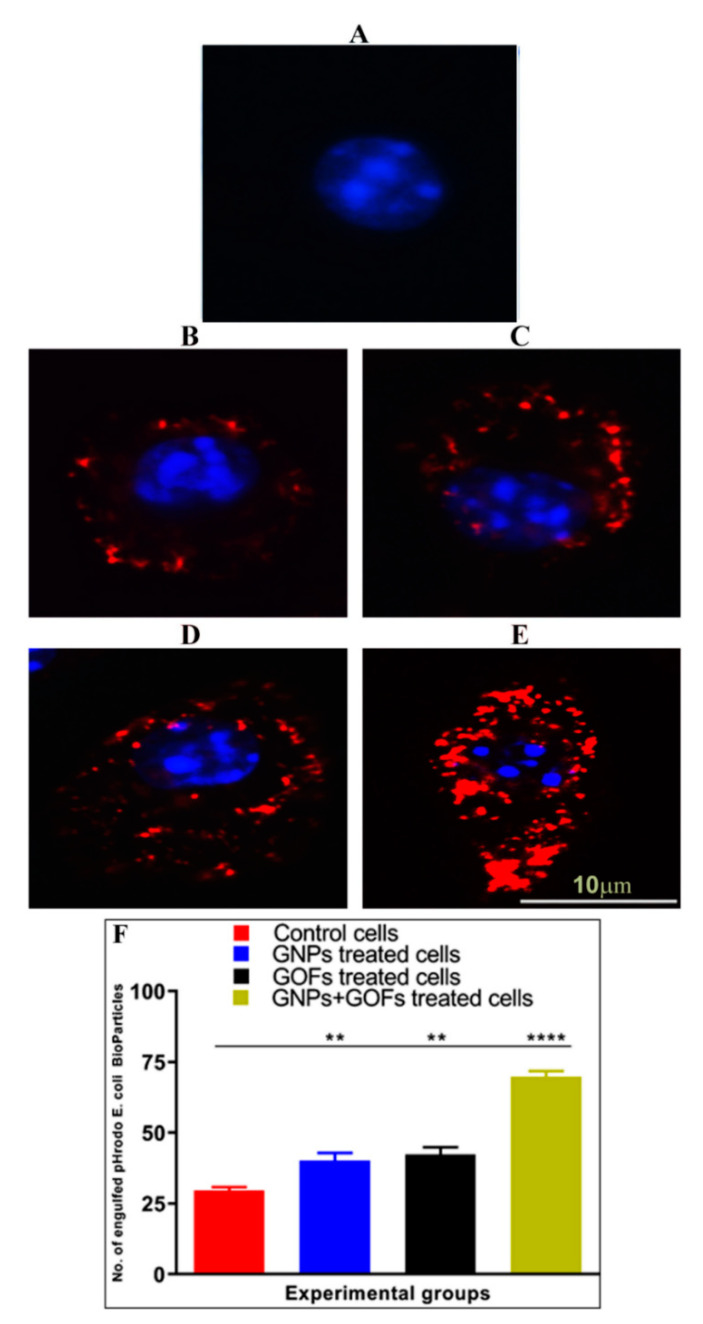
GNPs and GOFs enhanced phagocytosis of *pHrodo E. coli BioParticles*. (**A**) Control, untreated BMDMs cells stained with DAPI (blue). (**B**) Cells treated with *pHrodo E. coli BioParticles* (red) alone. (**C**) GNP-pretreated cells at a concentration of 10 µg/mL then treated with *pHrodo E. coli BioParticles.* (**D**) GOF-pretreated cells at a concentration of 10 µg/mL then treated with *pHrodo E. coli BioParticles*. (**E**) GNP- and GOF-pretreated cells at a 10 µg/mL concentration each then treated with *pHrodo E. coli BioParticles*. (**F**) The graph represents the number of engulfed of *pHrodo E. coli BioParticles*. Cells were quantified in 10 fields using Image J software version 1.43 (U.S. National Institutes of Health, Bethesda, MD, USA). Data are represented as mean ± SE. Statistical significance by one-way ANOVA (*p* < 0.0001) followed by posthoc testing using Tukey’s multi group comparison with control cells: ** *p* < 0.01, **** *p* < 0.0001 indicate statistically different from control BMDMs.

The phagocytic activity of BMDMs was determined by monitoring the uptake of tagged *E. coli* that showed a fluorescent signal only upon their trafficking into an acidic compartment of the lysosome (Figure 6A–E). A comparison of phagocytosis of *pHrodo E. coli BioParticles* followed by pretreatment of BMDMs with GNPs and GOFs was carried out. The results showed that the control BMDMs cells had less phagocytosis potential than the BMDMs cells, which were pretreated with GNPs and GOFs, Figure 6F.

### 3.6. GNPs and GOFs Induce NOX2 Function in BMDMs

**Figure 7:**
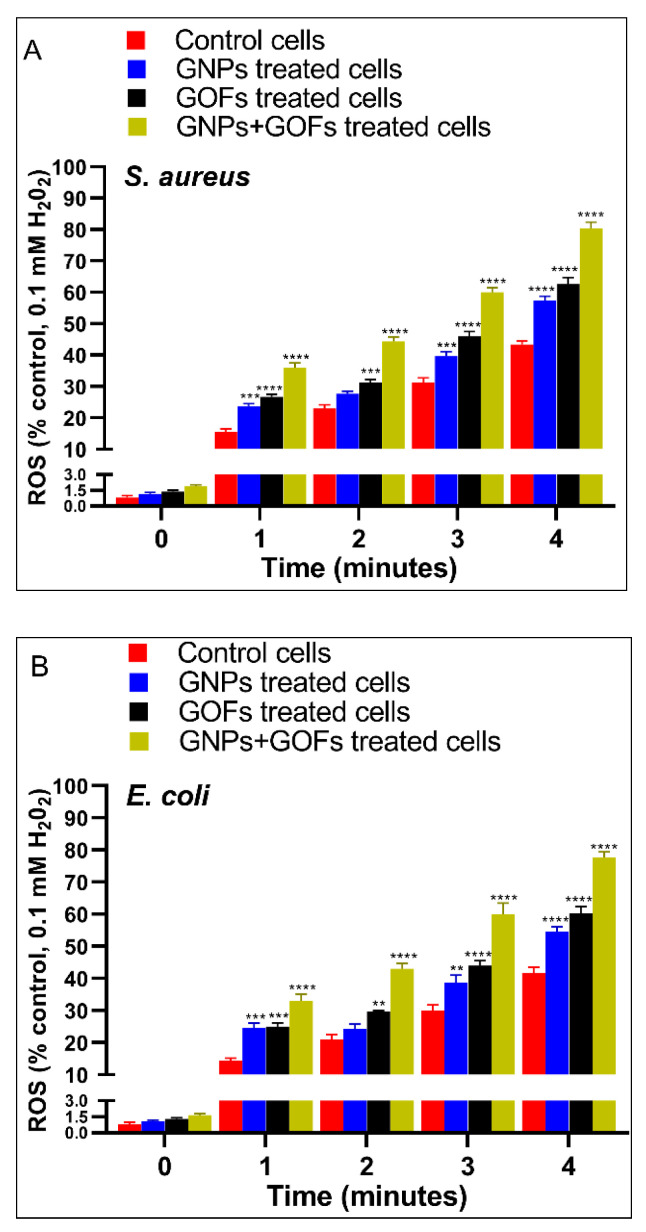
GNPs and GOFs increase reactive oxygen species (ROS) responses to bacterial strains in BMDMs. BMDMs were pre-treated with GNPs, and GOFs were then infected with *Staphylococcus aureus* or *Escherichia coli*. ROS was measured using Ampliflu. Kinetic readings of representative data are shown as mean ± SE at the indicated time. Statistical significance by two-way ANOVA (*p* < 0.0001) followed by posthoc testing using Tukey’s multi group comparison with control cells: *** *p* = 0.001, **** *p* < 0.0001 indicate statistically different for (**A**) *S. aureus* while ** *p* = 0.002, *** *p =* 0.0003, **** *p* < 0.0001 indicate statistically different for (**B**) *E. coli*.

**Figure 8:**
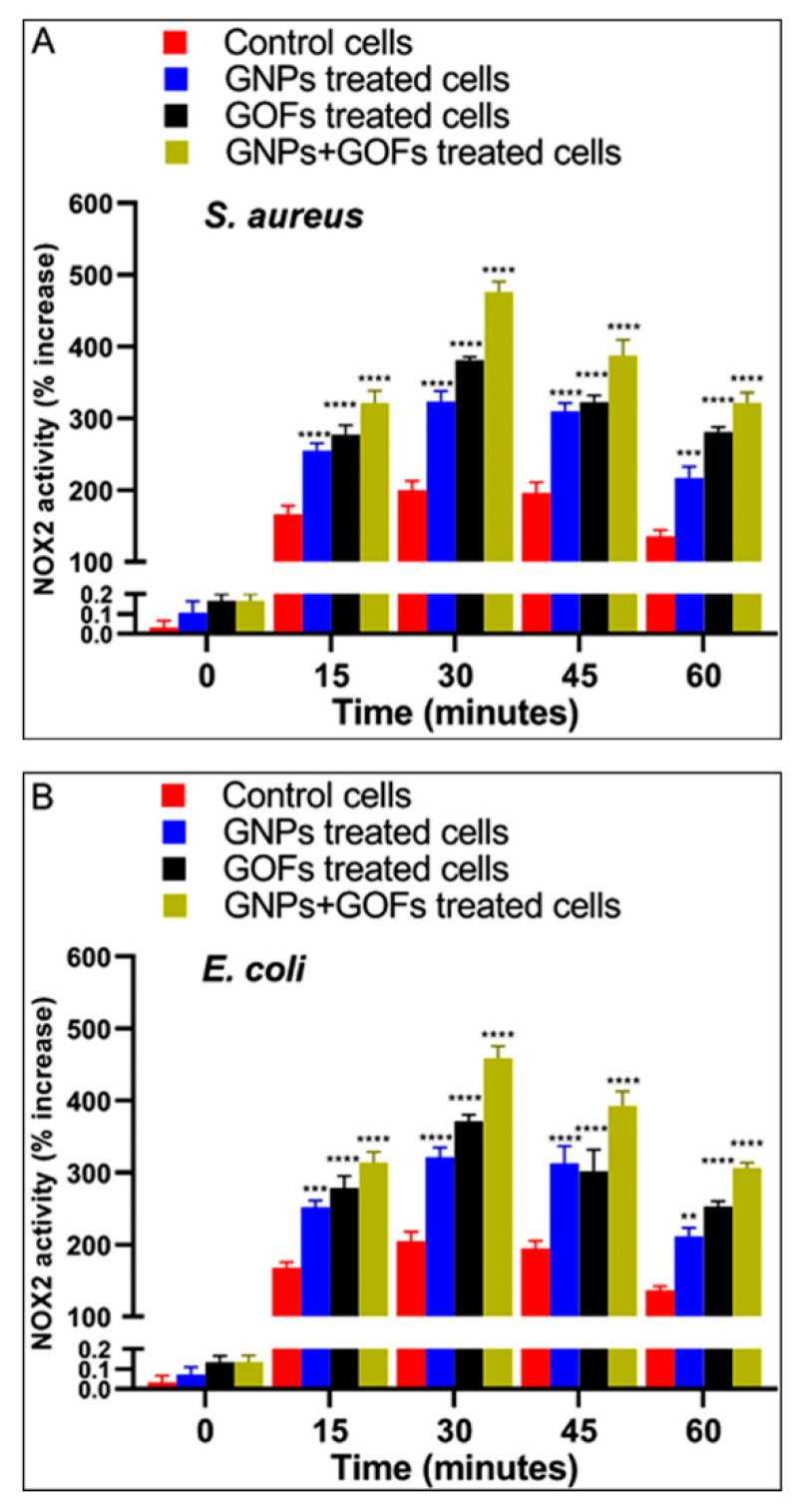
GNPs and GOFs improved NADPH oxidase 2 (NOX2) activity in BMDMs infected with *Staphylococcus aureus* or *Escherichia coli* in the presence and absence of GNPs and GOFs. NOX2 activity in BMDMs was assessed kinetically with lucigenin. Data are shown as mean ± SE at the indicated time. Statistical significance by two-way ANOVA (*p* < 0.0001) followed by posthoc testing using Tukey’s multi group comparison with control cells: *** *p* = 0.0001, ****p < 0.0001 indicate statistically different for (**A**) *S. aureus* while ** *p* = 0.002, *** *p =* 0.0004, ****p < 0.0001 indicate statistically different for (**B**) *E. coli.*

The phagocytes were able to kill the ingested bacteria through the crucial mechanism of the activation of the NOX2 enzyme complex, leading to ROS production (reactive oxygen species), i.e., superoxide anions. In the membranes of the phagosomes, these bacterial anions are produced by the activity of the NADPH oxidase complex NOX2, thereby leading the phagocytes to undergo more active killing of the ingested bacteria [59,60]. To test the NOX2 and ROS activities in the phagocytes, superoxide anion production by murine BMDMs was assayed. Compared with control BMDMs, the GNP- and GOF-pretreated BMDMs showed increased ROS and NOX2 activities, supposedly with the substantial release of superoxide anions in response to either *S. aureus* or *E. coli* presence (Figure 7 and Figure 8). Thus, the pretreatment of BMDMs with GNPs and GOFs was associated with increased NOX2 activity in response to improved ROS production, which is possibly one of the principal mechanisms leading to enhanced killing clearance of bacteria [61]. However, other mechanisms cannot be ruled out, since cell wall damage [62] and DNA/RNA destruction [63] was not monitored. In most probability, bacteria trappings in the aggregated GOFs and cell wall damage by the sharp edges of the GOFs pieces cannot be ruled out [64,65]. The DNA/RNA damage needs further work to estimate the damage to the nucleic acid component.

## 4. Conclusions

A significant increase in phagocytic cell activity was observed after introducing GNPs and GOFs in the experimental set-up studying cellular behaviour. The incremental phagocytosis activity was linked to the ROS and NOX2 pathway feedback. Presumably, the presence of immune-modulator entities to the involvement of increased levels of phagocytic cells, together with the presence of GNPs and GOFs leading to maturation of the phagocytic cells and involvement of the NOX2 pathway is a plausible explanation of the increased immune response and phagocytosis activity, which has the potential to be utilized for better antibacterial designs and preparation. The study also indicated that GNPs and GOFs could contain chemical components that act as immune-enhancers to increase phagocytic cell activity to engulf bacteria and other xenobiotics. However, the nanoparticles’ exact chemical roles need to be ascertained in further detail, and the chemical aspect of the interaction needs exploration. Taken together, the data demonstrated that GNPs and GOFs contribute to the two main bactericidal processes represented by the maturation of phagosomes and the production of ROS derived from NOX2 activation in this scenario. It can be concluded that the GNPs and GOFs are important contributors to the amplification of antibacterial responses initiated by the host’s innate immune system.
